# Commentary on “Acute pain service for postoperative pain in adults: a network meta-analysis”

**DOI:** 10.1097/JS9.0000000000003132

**Published:** 2025-08-06

**Authors:** Chunjuan Zhang, Runbing Xu, Jiamei Wen

**Affiliations:** a School of Life Sciences, Beijing University of Chinese Medicine, Beijing, China; b Department of Hematology and Oncology, Beijing University of Chinese Medicine Affiliated Dongzhimen Hospital, Beijing, China; c Pain Management Department, Xian Ning Ma Tang Hospital of Traditional Chinese Medicine, Xian Ning, Hubei, China

*Dear Editor,*

We read with great interest the recent network meta-analysis by Cheng and colleagues,” Acute Pain Service for Postoperative Pain in Adults: A Network Meta-Analysis”[1]. It systematically evaluated 38 randomized controlled trials (encompassing 6413 patients) to compare various acute pain service (APS) models. It was found that all APS models outperformed traditional doctor-nurse pain care, with the nurse-based anesthesiologist specialist-supervised APS (NBASS-APS) being the most effective. This provides valuable clinical insights for optimizing postoperative pain management. However, when converting these findings into clinical practice guidelines, a number of important constraints need to be carefully taken into account, especially with regard to methodological rigor, results’ generalizability, and clinical relevance. This correspondence is compliant with the TITAN Guidelines 2025 – governing declaration and use of AI[2].

Firstly, the study does not compare reported standardized mean differences (SMD) in pain reduction with established minimal clinically important differences (MCID). This makes it tough to determine the exact correlation of pain alleviation. As a result, while statistically significant, it may have no clinical relevance if the MCID is not met[3].

This omission significantly increases the risk of “spin” or overinterpretation of results, posing a major limitation that undermines the clinical interpretability and practical utility of the findings.

Secondly, comprehensive evaluation of APS extends beyond pain score reduction to encompass a broad spectrum of patient-centered, clinical, safety, and economic outcomes[4]. Although Table 1 indicates that some included studies reported parameters such as patient satisfaction, adverse events/postoperative complications, and opioid consumption, it fails to provide specific analysis. Furthermore, APS effectiveness is not solely determined by the service delivery model but is also significantly influenced by contextual factors including hospital tier, staffing ratios, and the training level of nursing and medical staff. The study’s heavy reliance on single-center data from China, without accounting for these institutional variables through reporting or statistical control, substantially limits the generalizability of its findings across diverse healthcare systems.

Thirdly, for studies reporting multiple postoperative time points, the meta-analysis selectively utilized only the “most commonly reported time point” VAS/NRS values. However, the actual evaluation time points varied substantially across included studies, which may have inadvertently introduced a selection bias in outcome reporting at the meta-analytic level – a phenomenon we term “time-point bias.”

Fourth, although the authors acknowledged heterogeneity and conducted subgroup analyses and meta-regression, these adjustments were limited to surgical type and patient age. Notably, anesthesia methods (including general anesthesia, neuraxial anesthesia, and local anesthesia) significantly influence postoperative pain perception. However, the included studies failed to report the distribution of anesthesia techniques, and the meta-analysis did not adjust for or perform subgroup analyses based on this critical confounding factor.

In conclusion, while this meta-analysis yields valuable statistical insights into treatment efficacy, the generalizability of its findings across diverse clinical settings remains questionable. Methodological shortcomings – including unaccounted biases, a single-country patient cohort, and an exclusive focus on short-term outcomes without MCID benchmarking – may impede direct clinical translation. Moving forward, rigorously designed, patient-centered studies are needed to comprehensively evaluate APS effectiveness across the entire recovery trajectory, thereby facilitating its broader clinical implementation

## Data Availability

All data are included in the article.
